# The effect of oral administration of NXP032 equivalent to intraperitoneal administration in an Alzheimer’s disease model

**DOI:** 10.3389/fphar.2025.1632640

**Published:** 2025-07-23

**Authors:** Jae Min Lee, You Jung Choi, Da-Eun Sung, Tae Hyeok Sim, So Hee Kim, Seung Geun Yeo, Youn-Jung Kim

**Affiliations:** ^1^ College of Nursing Science, Kyung Hee University, Seoul, Republic of Korea; ^2^ Department of Otorhinolaryngology Head & Neck Surgery, College of Medicine, Kyung Hee University Medical Center, Seoul, Republic of Korea; ^3^ Department of Nursing, Graduate School, Kyung Hee University, Seoul, Republic of Korea

**Keywords:** Alzheimer’s disease, 5xFAD, amyloid beta plaques, neuroinflammation, NXP032

## Abstract

**Introduction:**

Alzheimer’s disease (AD) is a progressive neurodegenerative disorder characterized by cognitive impairment, amyloid beta (Aβ) plaque accumulation, neuroinflammation, and neurodegeneration. Excessive oxidative stress exacerbates these pathologies, potentially accelerating disease progression. Although antioxidants like vitamin C can mitigate neuroinflammation and offer neuroprotective effects, their efficacy is often limited due to rapid oxidation, particularly when administered orally. NXP032 has been developed to stabilize vitamin C and sustain its antioxidant effects over time.

**Methods:**

This study evaluated the effects of NXP032 administered orally (PO) and intraperitoneally (IP) on AD pathology, specifically focusing on Aβ accumulation and neuroinflammation, using the 5xFAD mouse model over an 8-week period.

**Results:**

Both IP and PO administration of NXP032 significantly reduced Aβ plaque accumulation and thioflavin-S staining, while attenuating neuroinflammation in 5xFAD mice. This was associated with decreased neurodegeneration, evidenced by reduced Fluoro-Jade C staining in the hippocampus. Additionally, both administration routes resulted in significant cognitive function improvements.

**Discussion:**

NXP032 demonstrates potential as a therapeutic strategy to address multiple aspects of AD pathology and slow disease progression. The efficacy of oral administration is particularly notable, offering a practical method for clinical application.

## 1 Introduction

Alzheimer’s disease (AD) is a progressive neurodegenerative disorder and a leading cause of dementia, characterized by gradual memory loss and cognitive decline (Kumar et al., 2025). This deterioration primarily affects cognitive function and behavior, impairing the ability to perform daily activities and resulting in significant social and economic challenges (DeTure and Dickson, 2019). The pathogenesis of AD remains unclear, but its primary pathological features include the accumulation of amyloid-beta (Aβ) plaques, neurofibrillary tangles due to tau protein hyperphosphorylation, chronic neuroinflammation, synaptic dysfunction, and oxidative stress (Migliore et al., 2005; Duyckaerts et al., 2009). Aβ peptides are generated through the abnormal cleavage of amyloid precursor protein. Elevated levels of Aβ lead to the extracellular accumulation of aggregated Aβ in the brain (Bharadwaj et al., 2009). This increase in oxidative stress promotes Aβ deposition or Aβ fibril formation, which subsequently results in intracellular Aβ accumulation (Drake, 2003; Cheignon et al., 2018). Therefore, reducing oxidative stress may be an important therapeutic strategy for Alzheimer’s disease.

Oxidative stress is recognized as a critical pathological factor in AD, profoundly influencing disease progression (Tönnies and Trushina, 2017). It occurs when there is an imbalance between the production of reactive oxygen species (ROS) and the body’s ability to detoxify these reactive intermediates or repair the resulting damage (Chen et al., 2012). In AD, Aβ plays a pivotal role in contributing to oxidative stress by promoting the generation of ROS (Cheignon et al., 2018). These reactive molecules activate microglia, the brain’s resident immune cells, which leads to chronic neuroinflammation (Lull and Block, 2010). This inflammatory response exacerbates neuronal damage and triggers a cascade of pathological events. Furthermore, ROS influence cellular pathways by increasing caspase activity, which initiates apoptosis, or programmed cell death, thereby contributing to neuronal loss. Targeting ROS is a promising therapeutic strategy, offering the potential to alleviate oxidative stress and its downstream effects on Aβ pathology (Jomova et al., 2023). Additionally, ROS regulate kinases that are involved in the hyperphosphorylation of tau protein, thereby promoting the formation of neurofibrillary tangles, another hallmark of AD pathology (Zhang Y. et al., 2023). Targeting oxidative stress presents a promising therapeutic strategy for AD, with the potential to alleviate its detrimental effects on neuronal health and cognitive function (Tönnies and Trushina, 2017). Oxidative stress arises from an imbalance between the production of free radicals and the body’s ability to counteract their harmful effects through neutralization by antioxidants (Jomova et al., 2023). This imbalance contributes to the pathogenesis of AD by promoting neuronal damage, including the dysfunction of proteins like tau and Aβ (Zhang Y. et al., 2023). Thus, antioxidants may play a role in slowing the progression of AD and reducing neurodegeneration. This approach underscores the importance of addressing oxidative stress as a critical factor in the development and management of AD.

Vitamin C (ascorbic acid) is an essential micronutrient with antioxidant properties, capable of alleviating oxidative stress by scavenging ROS (Levine et al., 1999; Feng and Wang, 2012). It also plays a neuroprotective role, essential for maintaining neuronal function (Figueroa-Méndez and Rivas-Arancibia, 2015). However, vitamin C is easily oxidized in the body, limiting its stability as an antioxidant. To address this, a DNA aptamer (Aptamin C) that specifically binds to vitamin C has been developed (Choi et al., 2020). Aptamers are single-stranded nucleic acids that specifically bind to target molecules (Dunn et al., 2017), and Aptamin C enhances the stability of vitamin C. In our previous study involving NXP031, which was administered intraperitoneally in a 5xFAD animal model, we demonstrated its ability to reduce Aβ levels and improve cognitive function (Ju et al., 2025). Building on this, NXP032 is a novel compound that combines vitamin C with the DNA aptamer Aptamin® C320. This combination used in this study stabilizes vitamin C, preventing its rapid oxidation and extending its antioxidant effects. NXP032 has shown promise in counteracting oxidative stress by maintaining stable levels of vitamin C in the body until it reaches the target cells. Our previous research indicated that oral administration of NXP032 activates the Nrf2-ARE pathway, which enhances the expression of antioxidant enzymes and alleviates cognitive decline associated with aging (Lee et al., 2022; Lee et al., 2023). Despite these promising findings, the comparative efficacy of intraperitoneal versus oral administration of NXP032 in AD, particularly concerning Aβ accumulation and neuroinflammation, has not yet been fully explored.

In the present study, we investigated the effects of both intraperitoneal and oral administration of NXP032 on Aβ plaque accumulation, neuroinflammation, and cognitive function in the 5xFAD mouse model of AD. The 5xFAD transgenic mice are a well-established model for AD research, exhibiting key pathological features of the disease. We assessed the impact of NXP032 on cognitive function through behavioral tests and evaluated Aβ plaque accumulation in the cerebral cortex and hippocampus, along with markers of neuroinflammation. By comparing the efficacy of intraperitoneal versus oral administration, we aim to identify the most effective delivery method for NXP032.

## 2 Materials and methods

### 2.1 Animals and administration

The 5xFAD mice are transgenic mice that overexpress the human APP gene with Swedish, Florida, and London mutations, and the human PS1 gene with M146L and L286V mutations. This model rapidly develops amyloid beta (Aβ) accumulation and is commonly used as an Alzheimer’s disease model (Oakley et al., 2006). Professor Minho Moon from Konyang University, Daejeon, Republic of Korea, provided the male 5xFAD and wild-type mice, each weighing 28 ± 2 g and aged 10 months, for this study. The animals were housed 4 to 5 per cage, with free access to food and water, and maintained under controlled light/dark cycles (12 h light and 12 h dark) at a temperature of 22°C ± 2°C and 50% humidity. At 10 months of age, NXP032 was administered for 8 weeks, with dosing occurring every other day. The stabilized animals were divided into the following four groups: the normal wild-type group (WT + vehicle, n = 5), the 5xFAD transgenic group (5xFAD + vehicle, n = 5), the 5xFAD mice receiving intraperitoneal NXP032 (5xFAD + NXP032 IP, n = 4), and the 5xFAD mice receiving oral NXP032 (5xFAD + NXP032 PO, n = 4). Mice were treated with the same concentration of NXP032 (200 mg/kg ascorbic acid and 4 mg/kg Aptamin C320) via different administration methods to confirm the effect of oral administration. The experimental procedures adhered to the ethical policies of the institution and the guidelines for animal experiments established by the National Institutes of Health (NIH). All researchers involved in the study completed ethical training related to animal handling, injection, blood collection, and anesthesia administration, conducting the experiments with consideration for animal welfare. This study followed the guidelines for the management and use of laboratory animals of the NIH and was approved by the Institutional Animal Care and Use Committee of Kyung Hee University (KHSASP-20-568).

### 2.2 NXP032 preparation

NXP032 was prepared by dissolving the purified DNA aptamer, Aptamin® C320, provided by Nexmos Co., Ltd. (Yongin, Republic of Korea), in distilled water. The solution was heated to 95°C for 5 min to denature the DNA and then allowed to slowly cool to room temperature, facilitating the proper formation of its tertiary structure. The Aptamin® C320 was then mixed with ascorbic acid (Sigma-Aldrich, MO, United States) in a 1:50 weight ratio. Specifically, this resulted in concentrations of 4 mg of Aptamin® C320 and 200 mg/kg of ascorbic acid in the final NXP032 formulation. The pH of the NXP032 solution was adjusted to 5.7 to ensure stability.

### 2.3 Y-maze test

The Y-maze test was utilized to assess cognitive impairment associated with Alzheimer’s disease. The Y-maze consists of three arms arranged at 120-degree angles. The experiment was conducted over 2 days. On the first day, the experimental animals were placed in the center of the maze to explore each arm freely. On the second day, their behavior was evaluated over a 5-minute period. The test records whether the animals alternately visit new arms without repeatedly visiting the same arm, and this behavior is assessed as the alternation rate. The formula for calculating the spontaneous alternation rate is as follows:

$$\mathrm{The alternation rate}\left(\%\right)=\left[\left(\mathrm{No}.\mathrm{of alternations}\right)\;/\;\left(\mathrm{Total arm entries}-2\right)\right]\times 100.$$

### 2.4 Preparation of brain tissue

At the end of the experiment, animals were anesthetized using ethyl ether inhalation (5 mL/250 g). Once sufficiently anesthetized, the thoracic cavity was opened to expose the heart, and a 24G needle was inserted into the left ventricle for perfusion with phosphate-buffered saline (PBS) to remove blood. Subsequently, perfusion with 4% paraformaldehyde (PFA) was performed to fix the tissues, and the brain was extracted. The extracted brain was immersed in 4% PFA at 4°C for an additional 24 h to ensure thorough fixation. Following fixation, the brain tissue was dehydrated in a PBS-diluted 30% sucrose solution. Once adequately dehydrated, the brain was sectioned coronally at a thickness of 30 µm using a cryostat (Leica, Nussloch, Germany).

### 2.5 Thioflavin-S staining

Thioflavin-S staining is a histological method used to detect amyloid plaques by specifically binding to the β-sheet structures of amyloid. To begin, Thioflavin-S (Th-S, Sigma-Aldrich, St Louis, MO, United States) is dissolved in 50% ethanol to create a 0.5% solution. The brain tissue is then washed with PBS and incubated in the 0.5% Th-S solution for 10 min at room temperature, taking care to avoid direct sunlight exposure. After staining, the tissue is washed with 50% ethanol followed by PBS. The Th-S stained brain tissues are subsequently mounted on gelatin-coated slides using VECTASHIELD® with DAPI and cover-slipped. Images of the stained tissues are captured using a confocal microscope (Zeiss LSM 700; Zeiss; Oberkochen, Germany) and analyzed with ImageJ software (Bethesda, MD, United States).

### 2.6 Fluoro-Jade C staining

For Fluoro-Jade C (FJC) staining, brain section slides are initially incubated in a solution containing 1% NaOH and 80% ethanol for 5 min. Following this, the slides are rinsed in 70% ethanol and then in distilled water for 2 min each. The sections are subsequently immersed in a 0.06% potassium permanganate solution for 20 min. After a rinse with distilled water, the sections are incubated in a 0.0004% FJC solution, which is prepared in 0.1% acetic acid, for 20 min. After several washes, the sections are treated with DAPI for an additional 20 min. The slides are then thoroughly dried in a shaded environment and mounted using DPX mounting medium. The number of FJC-positive cells in the hippocampus is quantified through stereological counting using ImageJ software (Bethesda, MD, United States).

### 2.7 Immunohistochemistry and immunofluorescence

For immunohistochemical analysis, sections of brain tissue, specifically from the hippocampal region, were selected and washed three times with 0.05M PBS. To prevent non-specific antibody binding, endogenous peroxidase (HRP) activity was quenched by treating the tissues with a 3% hydrogen peroxide (H_2_O_2_) solution for 30 min, followed by three washes with PBS. Subsequently, a blocking step was performed at room temperature for 2 h using a 1% bovine serum albumin (BSA) solution mixed with serum from the same species as the secondary antibody host. After blocking, the sections were washed three times with PBS and incubated overnight at 4°C with primary antibodies: anti-Amyloid beta (1:1,000, ab201060, Abcam, Cambridge, United Kingdom), anti-Iba-1 (1:1,000, ab178846, Abcam, Cambridge, United Kingdom), and anti-GFAP (1:1,000, PA3-16727, Invitrogen, Thermo Fisher Scientific, Waltham, MA, United States). Following incubation, the sections were washed three times with PBS and then incubated with secondary antibodies at room temperature for 1 h and 30 min. After another three PBS washes, the sections were reacted with an avidin-biotin complex (Vector Elite ABC kit, Vector Laboratories, Burlingame, CA, United States) for 1 h and 30 min. Color development was performed using 3,3-diaminobenzidine tetrahydrochloride (DAB kit, Vector Laboratories, Burlingame, CA, United States) for 2–5 min. The tissues were then mounted on slides and dried. The dried tissues underwent dehydration through a graded ethanol series (70%–80%–90%–100% for 3 min each, with 100% repeated twice), followed by clearing with xylene for 10 min. Finally, the sections were covered with coverslips using permount and observed under a light microscope (BX51, Olympus Co., Ltd., Tokyo, Japan) at 10X and 40X magnifications to examine the hippocampus and cerebral cortex. The area (%) of immunoreactive regions, indicating Aβ aggregation and microglial and astrocytic activation, was analyzed using the ImageJ program (Bethesda, MD, United States). All images were analyzed blindly.

For immunofluorescence, the sections were initially washed with 0.05 M PBS, followed by incubation in a blocking solution containing 2% BSA and 10% normal goat serum in 0.05 M PBS for 2 h at room temperature. Subsequently, the sections were incubated overnight at 4°C with primary antibodies targeting Aβ (1:500; ab201060, Abcam) and CD11b (1:500; LS-C62935, LifeSpan BioSciences, United States). Following PBS washing, the sections were treated with a mixture of Alexa Fluor 488-conjugated donkey anti-rat IgG (1:1,000; Molecular Probes, Eugene, OR) and Alexa Fluor 594-conjugated goat anti-rabbit IgG (1:1,000; Molecular Probes, Eugene, OR) for 1 h at room temperature. Images were captured using a Zeiss LSM 700 confocal microscope (Oberkochen, Germany).

### 2.8 Statistical analysis

All experimental data are expressed as means ± standard error of the mean (SEM). Statistical analyses were conducted using SPSS software (version 25.0; IBM SPSS, Chicago, IL, United States) or GraphPad Prism software (version 8.0; GraphPad Software Inc., San Diego, CA, United States). A one-way analysis of variance (ANOVA) was utilized to assess differences between groups. For post-hoc analysis, Tukey’s honestly significant difference test was employed, with statistical significance determined at p < 0.05.

## 3 Results

### 3.1 NXP032 enhances cognitive function in 5xFAD mice

**FIGURE 1:**
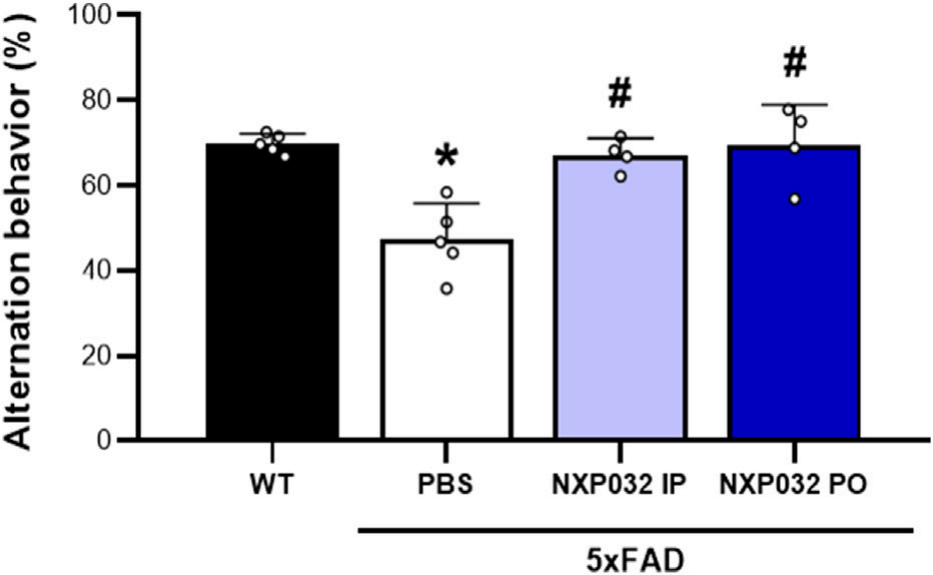
Effects of intraperitoneal (IP) and oral (PO) administration of NXP032 on cognitive impairment in Alzheimer’s Disease (AD), as assessed by the Y-Maze test. Treatment with NXP032 significantly increased spontaneous alternation behavior compared to PBS treatment. Data are presented as mean ± standard error of the mean (SEM) for each group: Wild Type (WT, n = 5), PBS (n = 5), NXP032 IP (n = 4), and NXP032 PO (n = 4). Statistical analysis was performed using one-way ANOVA followed by Tukey’s post hoc test. *p < 0.05 compared to the WT group. #p < 0.05 compared to the 5xFAD + PBS group.

The assessment of cognitive function using the Y-maze test revealed significant differences in the alternation rate (%) among the experimental groups. The 5xFAD + PBS group exhibited a significantly reduced alternation rate compared to the wild-type (WT) group (F_3,14_ = 13.027, p < 0.001), indicating cognitive impairment due to Alzheimer’s disease (AD). In contrast, both the intraperitoneally and orally administered NXP032 groups showed a significantly increased alternation rate compared to the vehicle group (p < 0.001). This finding suggests that NXP032 effectively improves cognitive impairment. Furthermore, there was no significant difference in the alternation rate between the intraperitoneal (IP) and oral (PO) administration groups, indicating that the route of administration does not significantly influence the cognitive recovery effect of NXP032 (Figure 1).

### 3.2 Reduction of Aβ plaque accumulation by NXP032 administration in 5xFAD mice

**FIGURE 2:**
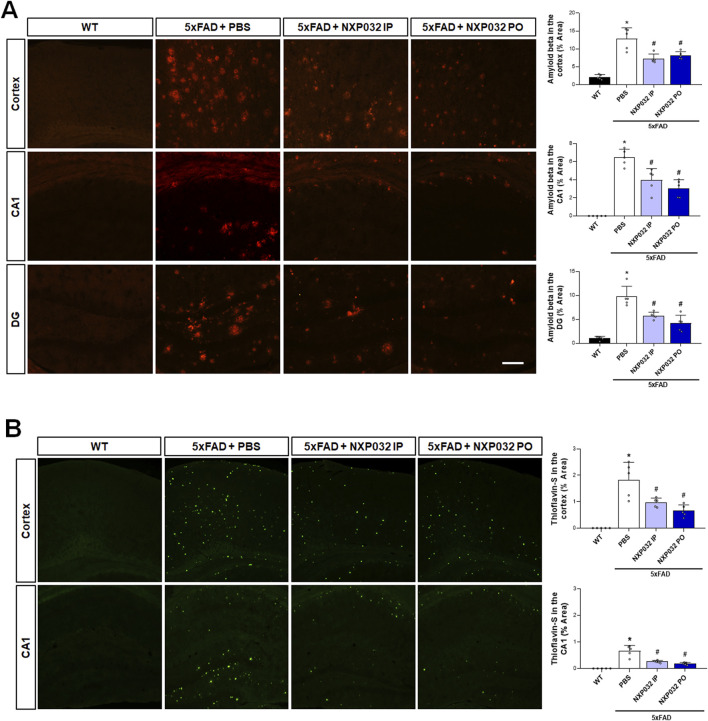
Effects of IP and PO administration of NXP032 on Aβ plaque accumulation in the hippocampus and cerebral cortex of 5xFAD mice. **(A)** Representative microscopic images of Aβ immunohistochemistry. **(B)** Representative images of Thioflavin-S staining. The graphs on the right display the quantified data. Thioflavin-S and Aβ accumulation were markedly reduced with both intraperitoneal and oral administration of NXP032 in both brain regions. Scale bar = 200 μm. Data are presented as mean ± standard error of the mean (SEM) for each group: Wild Type (WT, n = 5), PBS (n = 5), NXP032 IP (n = 4), and NXP032 PO (n = 4). Statistical analysis was performed using one-way ANOVA followed by Tukey’s post hoc test. Data are presented as mean ± standard error of the mean (SEM). *p < 0.05 compared to the WT group. #p < 0.05 compared to the 5xFAD + PBS group.

To examine Aβ plaque accumulation, anti-Aβ antibody staining and Thioflavin-S histochemistry and immunohistochemistry were performed on the hippocampus and cerebral cortex using specific antibodies for Aβ accumulation. The 5xFAD mice treated with NXP032 showed a significant reduction in Aβ accumulation areas in the hippocampal CA1 region (F_3,17_ = 49.544, p < 0.001), the dentate gyrus (F_3,17_ = 31.544, p < 0.001), and the cerebral cortex (F_3,17_ = 50.138, p < 0.001) (Figure 2A). Thioflavin-S staining further demonstrated that both IP and PO administration of NXP032 significantly reduced Th-S positive areas in the hippocampal CA1 (F_3,17_ = 17.29, p < 0.001) and the cerebral cortex (F_3,17_ = 17.29, p < 0.001) (Figure 2B). These results suggest that both IP and PO administration of NXP032 can significantly reduce Aβ accumulation in the brains of 5xFAD mice. Although there was no statistically significant difference between the two routes of administration, PO administration resulted in slightly greater reductions in Thioflavin-S and Aβ accumulation.

### 3.3 Alleviation of neuroinflammation through NXP032 administration in 5xFAD mice

**FIGURE 3:**
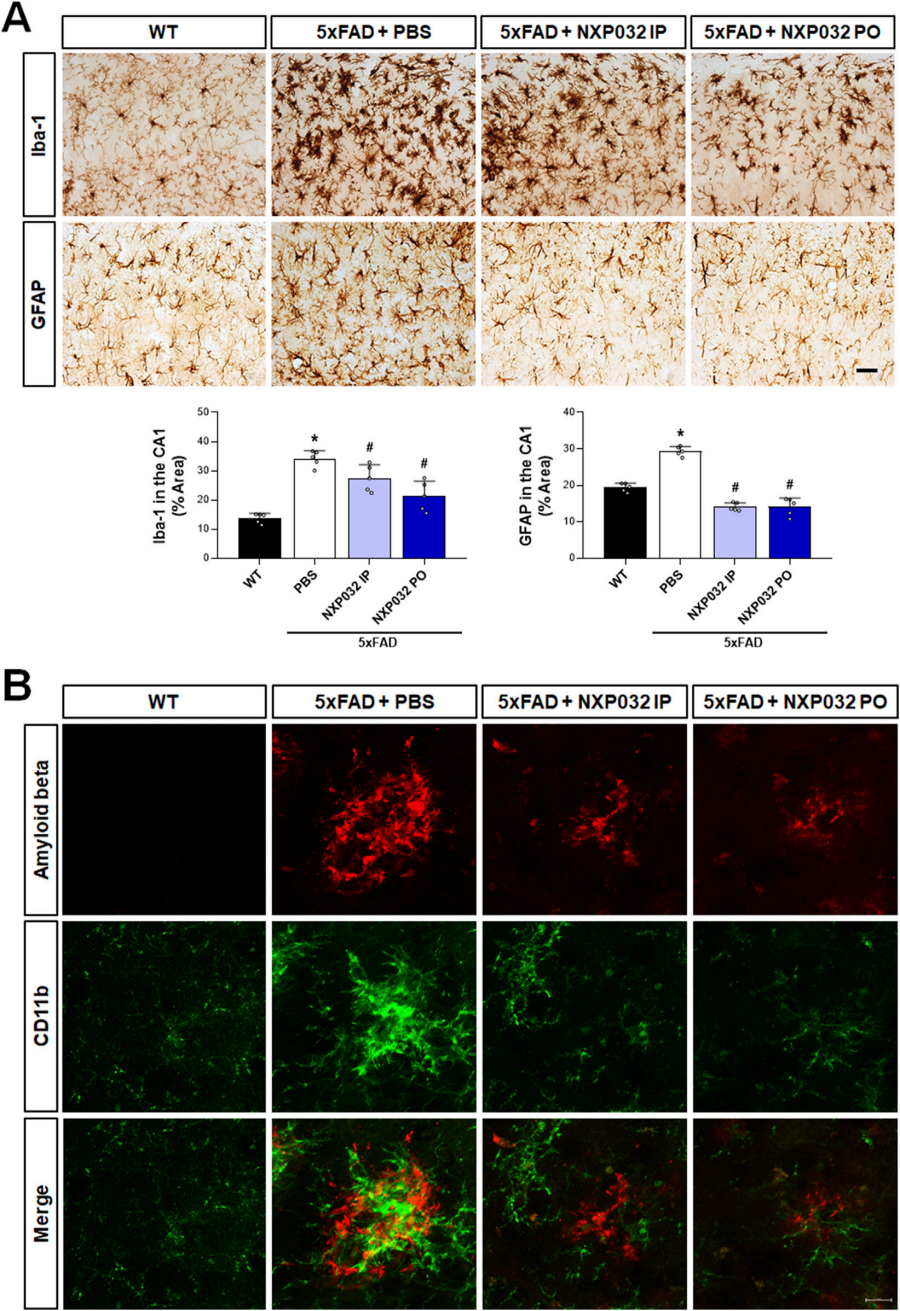
Effects of IP and PO administration of NXP032 on the activation of microglia and astrocytes in the hippocampal CA1 region. **(A)** Immunohistochemical analysis was performed to assess the reactivity of microglia and astrocytes in the hippocampus of 5xFAD mice using Iba-1 and GFAP. In 5xFAD mice, there was an observed increase in the activation of microglia and astrocytes, indicating heightened neuroinflammatory responses. Both the intraperitoneal and oral administration groups of NXP032 showed reduced activation of these cells. **(B)** In the cerebral cortex of 5xFAD mice, Aβ plaques were observed to be surrounded by activated microglia, as indicated by CD11b staining. Scale bar: **(A)** 50 μm and **(B)** 10 μm. Data are presented as mean ± standard error of the mean (SEM) for each group: Wild Type (WT, n = 5), PBS (n = 5), NXP032 IP (n = 4), and NXP032 PO (n = 4). Statistical analysis was performed using one-way ANOVA followed by Tukey’s post hoc test. *p < 0.05 compared to the WT group. #p < 0.05 compared to the 5xFAD + PBS group.

Toxic Aβ aggregates are well known for their role in inducing neuroinflammation. To assess whether NXP032 alleviates neuroinflammation in 5xFAD mice, we conducted immunohistochemical analyses using Iba-1 and GFAP to evaluate the reactivity of microglia and astrocytes in the hippocampal CA1 region. Our findings revealed that positive signals for Iba-1 and GFAP were significantly elevated in the 5xFAD group compared to the WT group, indicating activation of microglia and astrocytes. However, both IP and PO administration of NXP032 resulted in a significant reduction of Iba-1 (F_3,17_ = 30.849, p < 0.001) and GFAP (F_3,17_ = 106.165, p < 0.001) positive areas, suggesting a mitigation of neuroinflammatory responses (Figure 3A). In the cerebral cortex of 5xFAD mice, Aβ plaques were observed to be surrounded by activated microglia, as demonstrated by the co-localization of CD11b staining with Aβ plaques. This co-localization indicates active recruitment of microglia, marked by CD11b, to sites of Aβ deposition (Figure 3B). Remarkably, treatment with NXP032 led to a reduction in both Aβ plaque burden and microglial activation, highlighting its potential to ameliorate neuroinflammation in this Alzheimer’s disease model.

### 3.4 Reduction of neuronal degeneration in 5xFAD mice through NXP032 administration

**FIGURE 4:**
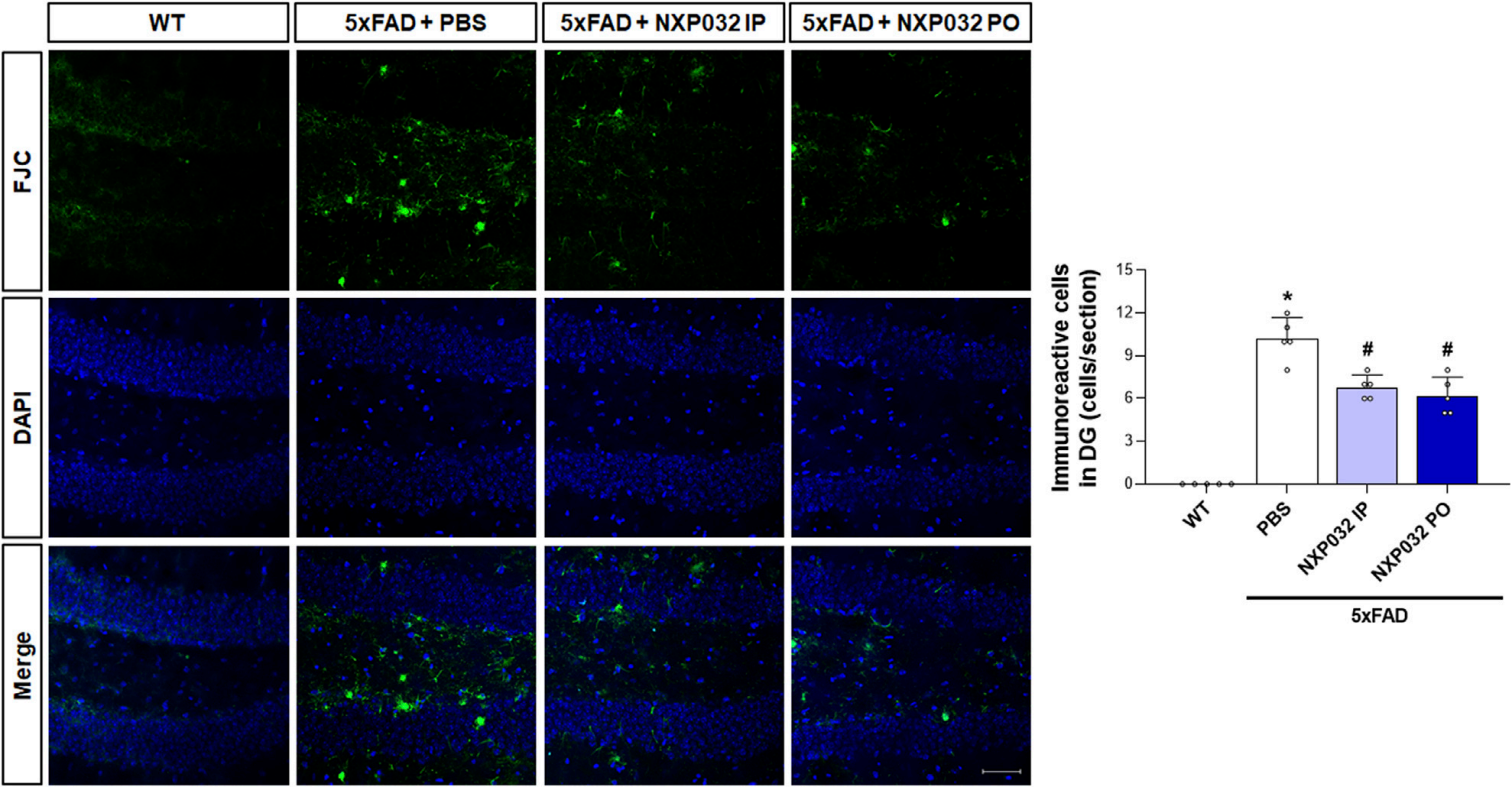
Effects of IP and PO administration of NXP032 on neuronal degeneration in the hippocampal dentate gyrus (DG). The representative photomicrographs show Fluoro-Jade C (FJC)-positive cells in the hippocampal DG. In 5xFAD mice, there was an increase in the number of FJC-positive cells, indicating heightened neuronal degeneration. However, both intraperitoneal and oral administration of NXP032 resulted in a reduction of FJC-positive cells, suggesting that NXP032 has a protective effect against neuronal degeneration in the 5xFAD mice model. Scale bar: 50 μm. Data are presented as mean ± standard error of the mean (SEM) for each group: Wild Type (WT, n = 5), PBS (n = 5), NXP032 IP (n = 4), and NXP032 PO (n = 4). Statistical analysis was performed using one-way ANOVA followed by Tukey’s post hoc test. *p < 0.05 compared to the WT group. #p < 0.05 compared to the 5xFAD + PBS group.

To investigate whether intraperitoneal and oral administration of NXP032 improves neuronal degeneration, we examined neuronal cell death markers in the hippocampal dentate gyrus (DG) region. Using Fluoro-Jade C (FJC) staining, which specifically and sensitively identifies degenerating neurons, we confirmed that the 5xFAD group exhibited greater neuronal degeneration in the hippocampal DG compared to the WT group. However, both intraperitoneal and oral administration of NXP032 led to a significant reduction in FJC-positive cells (F_3,17_ = 78.493, p < 0.001). This finding indicates that NXP032 exerts a protective effect against neuronal degeneration in this AD model (Figure 4).

## 4 Discussion

This study investigates the neuroprotective potential of NXP032 in an Alzheimer’s disease (AD) animal model characterized by amyloid-beta (Aβ) accumulation. We explored the therapeutic efficacy of NXP032 by comparing intraperitoneal (IP) and oral (PO) administration routes in 5xFAD mice. Our results demonstrate that both IP and PO administration of NXP032 significantly reduced Aβ accumulation and the activation of microglia and astrocytes in the cerebral cortex and hippocampus. These effects were associated with improved cognitive function, as evidenced by performance in the Y-maze test. Moreover, both administration methods markedly decreased thioflavin-S, Fluoro-Jade C (FJC), and Aβ immunoreactivity in the hippocampus and cerebral cortex, indicating a reduction in amyloid pathology. While oral administration exhibited slightly greater efficacy, the difference was not statistically significant. Our findings suggest that NXP032 may serve as a promising therapeutic agent for AD.

AD is significantly influenced by oxidative stress as a pathological factor. Aging, a major risk factor for AD, leads to the accumulation of oxidative and metabolic stress in the brain (Zhao and Zhao, 2013). Increased oxidative stress acts as an early event in AD pathology, inducing membrane damage, cytoskeletal changes, and apoptosis, while also being closely related to Aβ accumulation. (Tamagno et al., 2021). Aging-related oxidative stress can enhance the activity of β and γ secretases, enzymes responsible for cleaving amyloid precursor protein, thus leading to the overproduction of Aβ (Cheignon et al., 2018). Therefore, reducing oxidative stress is considered crucial in treating AD. In our recent previous study, NXP031 demonstrated potential as a therapeutic agent for Alzheimer’s disease by inhibiting amyloid-beta accumulation, reducing oxidative stress, and suppressing neuroinflammation, thereby ultimately protecting against memory impairment and neuronal damage (Ju et al., 2025). Furthermore, in an animal experiment on Parkinson’s disease, IP administration of NXP031 was found to prevent oxidative damage and protect dopaminergic neurons from degeneration by reducing oxidative stress markers (Song et al., 2022). Previous research has demonstrated that oral administration of NXP032, a vitamin C/DNA aptamer complex, effectively activates the Nrf2-ARE signaling pathway, thereby enhancing the expression of key antioxidant enzymes such as SOD-1 and GSTO1/2 (Lee et al., 2022). This activation plays a crucial role in mitigating oxidative stress and alleviating cognitive decline associated with aging. Although the current study does not directly measure Nrf2 activation, the observed neuroprotective effects of NXP032 in reducing Aβ plaque accumulation and improving cognitive function in 5xFAD mice could be attributed to this mechanism (Lee et al., 2022). By enhancing the cellular antioxidant response, NXP032 likely reduces oxidative damage and provides neuroprotection, consistent with our earlier findings. This suggests that NXP032 may serve as a potential therapeutic intervention for oxidative stress-related neurodegenerative conditions, including Alzheimer’s Disease. In models of degenerative diseases, including AD, the administration of NXP031 and NXP032 has demonstrated the ability to alleviate cognitive impairment by mitigating oxidative stress and neuroinflammation in the brain.

Oxidative stress can exacerbate neuroinflammation, a critical component of AD pathology, by activating microglia and astrocytes (Di Benedetto et al., 2022). This activation leads to the release of inflammatory cytokines, which further damage neurons. Aβ induces inflammatory responses by stimulating microglia, which play a crucial role in preventing Aβ deposition through phagocytosis (Gao et al., 2023). Microglial cells, which are immune cells in the brain, become activated in response to inflammation and play a crucial role in removing Aβ, although they are also activated by its accumulation. Studies on autopsy tissues from AD patients have shown increased microglial activation, particularly around Aβ plaques, which are characteristic of AD (Liu and Hong, 2003; Sastre et al., 2006). Activated microglia release cytokines and chemokines, some aiding Aβ removal and others worsening neuroinflammation and causing neuronal damage (Zhang W. et al., 2023). Additionally, astrocytes are induced to secrete neurotoxic substances and produce inflammatory cytokines in response to Aβ stimulation (González-Reyes et al., 2017). Although microglia can protect the brain by removing Aβ and releasing anti-inflammatory cytokines, chronic activation due to persistent Aβ accumulation may lead to sustained inflammation and contribute to neurodegeneration. Our results also showed hyperactivation of microglia and astrocytes and increased Aβ accumulation in 5xFAD mice. However, both IP and PO administration of NXP032 effectively suppressed neuroinflammation.

Therefore, reducing oxidative stress is crucial for slowing the pathological progression of AD, with vitamin C serving as an essential antioxidant in the brain. Notably, the average plasma vitamin C concentration in AD patients is approximately 30% lower than in control groups (Lopes da Silva et al., 2014), and cognitively healthy individuals tend to have higher vitamin C levels (Travica et al., 2017). This suggests that vitamin C deficiency may influence AD progression, and vitamin C supplementation could be a viable strategy for prevention and treatment. In a study involving 5xFAD mice, high-dose vitamin C administration was reported to reduce Aβ accumulation in the cerebral cortex and hippocampus, as well as alleviate mitochondrial dysfunction (Kook et al., 2014). These findings indicate that vitamin C supplementation may significantly reduce Aβ accumulation and improve cognitive impairment in AD patients. To maximize the neuroprotective effect of vitamin C, it is essential that it is delivered to the brain in a stable form, without undergoing oxidation within the body. Conventional oral administration results in a significant portion of vitamin C being decomposed in the small intestine, thereby reducing its bioavailability (Pérez-Vicente et al., 2002; Vallejo et al., 2004; Rodríguez-Roque et al., 2013). In this study, no significant difference was observed in the effects on cognitive impairment improvement, Aβ accumulation reduction, and neuroinflammation reduction between IP and PO administration of NXP032. This suggests that NXP032 can maintain sufficient antioxidant effects and protect vitamin C within the gastrointestinal tract even when administered orally. Therefore, NXP032 could offer a viable treatment option for AD with the convenience of oral administration. These results indicate that NXP032 can overcome the limitations of conventional vitamin C formulations, providing a promising treatment option through oral administration for future clinical applications.

In experimental animal studies, IP administration is a common method for drug delivery due to its relative simplicity and higher bioavailability compared to PO administration. IP administration is generally more efficient because it allows for direct absorption into the systemic circulation, bypassing the first-pass metabolism that reduces drug concentration during PO administration. This can lead to faster and more complete absorption, making IP an effective approach for evaluating drug efficacy in preclinical settings (Al Shoyaib et al., 2019). However, IP administration is rarely used in clinical practice due to potential risks such as infection and irritation; instead, PO administration is preferred for its convenience and higher patient compliance. Despite its advantages, PO administration may result in reduced bioavailability because of first-pass metabolism in the liver and variable absorption rates influenced by gastrointestinal pH and the physicochemical properties of the drug (Benet, 1978). For instance, vitamin C is primarily absorbed in the intestine via the sodium-dependent vitamin C transporter 1 (SVCT1) (Padayatty et al., 2004), but it is prone to rapid degradation due to high pH levels and digestive enzymes in the intestine (Pérez-Vicente et al., 2002; Rodríguez-Roque et al., 2013). In a previous study, Aptamin C, when administered following an intratracheal injection of bleomycin, was shown to significantly enhance the expression of sodium-dependent vitamin C transporters, SVCT-1 (Shin et al., 2024). This upregulation facilitates increased absorption and reabsorption of vitamin C across various tissues, thereby improving its bioavailability and therapeutic efficacy. Notably, SVCT1 is primarily involved in the absorption processes in the liver, kidneys, and intestines, while SVCT2 plays a critical role in maintaining vitamin C levels in the brain, eyes, heart, and lungs (Savini et al., 2008). For vitamin C to be effective, its concentration in organs is considered highly important, potentially even more so than its concentration in the blood (Kim et al., 2012). The concentration of vitamin C in various organs indicates that orally ingested vitamin C is absorbed through the digestive system and accumulates in these organs, where it is utilized as needed. The accumulation in the spleen, an immune organ, highlights the role of vitamin C in activating immune cells, including NK cells (Agura et al., 2024). Understanding the distinction between intraperitoneal and oral administration is crucial for assessing bioavailability and therapeutic efficacy of vitamin C. Intraperitoneal administration causes a rapid increase in blood concentration but often results in swift clearance, potentially limiting long-term efficacy due to insufficient organ accumulation. In contrast, oral administration allows for gradual absorption and sustained retention within the body. NXP032 appears to facilitate not only the effects of intraperitoneal administration but also enhances organ concentration through oral administration, promoting more sustained retention in the body.

The limitations of this study are as follows: First, we were unable to directly measure vitamin C levels in blood and brain tissue after 8 weeks of NXP032 administration. This limitation prevents us from fully understanding the compound’s pharmacokinetic profile. Therefore, we suggest that future pharmacokinetic studies on NXP032 are needed to support therapeutic equivalence of oral administration. Second, we could not clearly demonstrate differences in bioavailability between IP and PO administration. This gap highlights the need for future studies to investigate these pharmacokinetic parameters to better elucidate the differential effects of administration routes. Third, the limited sample size may affect the generalizability of our results. However, despite the small number of subjects, statistically significant results were obtained between groups, supporting the efficacy of NXP032. These findings offer valuable preliminary insights, and larger-scale studies are necessary to confirm and extend our observations. Fourth, we tested only a single dose of NXP032, which limits our understanding of the dose-response relationship and optimal dosing strategy. While we did not evaluate multiple doses, the observed effects at the single concentration, informed by the dosage used in our previous study, effectively demonstrate the efficacy of NXP032 (Lee et al., 2022; Lee et al., 2023). This dosage effectively modulated Aβ pathology, reducing Aβ accumulation and alleviating cognitive impairment, neuronal apoptosis, synaptic degeneration, and neuroinflammation caused by Aβ toxicity.

## 5 Conclusion

NXP032 demonstrated significant neuroprotective effects in an AD animal model by reducing Aβ accumulation and decreasing the activation of microglia and astrocytes, thereby improving cognitive impairment. Both IP and PO administration routes were effective, with no statistically significant differences observed between them. This suggests that oral administration of NXP032 can maintain its antioxidant effects and protect vitamin C in the gastrointestinal tract. These findings highlight NXP032 as a viable treatment option for AD, offering the convenience and practicality of oral administration. Furthermore, NXP032 has the potential to overcome the limitations of existing vitamin C formulations, indicating its promise as a therapeutic strategy for AD and other neurodegenerative diseases.

## Data Availability

The original contributions presented in the study are included in the article/supplementary material, further inquiries can be directed to the corresponding author.
